# Americal Dental Association

**Published:** 1869-09

**Authors:** 


### Editorial.

---

### AMERICAN DENTAL ASSOCIATION.

THE Ninth Annual Meeting of the "American Dental Association" was held at Saratoga, on the 3d, 4th, 5th and 6th of August.

A very pleasant time was enjoyed by all those present, both in and out of the meeting.

The sessions were equally as interesting and harmonious, if not more so, than those of any former years.

There were, perhaps, one hundred and seventy members present.

The character of the papers and discussions certainly show most clearly, that the active minds of the profession are very far from resting satisfied with present attainments.

There is great search being made into the deep things of science, and an appropriation of whatever may be used for the development and upbuilding of our profession. Judging from the multiplicity of instruments, appliances, methods and processes that were presented there, one must be fully impressed with the fact, that the inventive faculties of the profession are not soon to be exhausted.

There is a large number of men in the Dental profession who seem to be fully aware that there are yet unexplored fields, the cultivation of which will yield a rich reward. We hail with delight the success of the work, in both the art and science.

We most heartily approve the selection of Nashville, as the next place of meeting of the Association, and we hope there will be no Niagara there, with its attractive grandeur, nor Saratoga, with its ten thousand fascinations—races, are an attraction to some people. Twelve o'clock sometimes reduced the number in attendance at our meetings in a marked degree.

Nashville is surrounded with beauty, and full of it too, and we trust the members of our Association will rest so well satisfied of that fact, that they will not feel impelled to go in search of something magnificent or grand, but will be able fully to give their undivided attention to the work in hand.

One of the greatest obstacles to the progress of the Dental profession is found in the fact, that a very large proportion of its members, are not sufficiently in earnest. Were it not so, the American Dental Association would have at least one thousand active, earnest workers in attendance, and they so fully intent upon the accomplishment of the one great common object that they would move and work as one man.

The subject of professional education was almost wholly overlooked, which we regard as a great mistake. This Association being national, its counsels and declarations will necessarily exercise an influence upon the whole profession; and we are confident that no more profitable theme could occupy the attention of the Association than the education and preparation of those who are in the future to enter its ranks, and be its standard bearers. A great responsibility rests upon those now in the field in this respect; and the proper means of discharging this responsibility should receive most serious consideration. We trust this subject will receive more attention at the future meetings. T.

